# Relationships between gut microbiota, red meat consumption and colorectal cancer

**Published:** 2022-05-12

**Authors:** Mahamane Talphi Diakité, Bréhima Diakité, Amadou Koné, Saidou Balam, Djeneba Fofana, Dramane Diallo, Yaya Kassogué, Cheick B Traoré, Bakarou Kamaté, Djibril Ba, Madani Ly, Mamadou Ba, Bourahima Koné, Almoustapha I. Maiga, Chad Achenbach, Jane Holl, Robert Murphy, Lifang Hou, Mamoudou Maiga

**Affiliations:** 1 University of Sciences, Techniques and Technologies of Bamako (USTTB), Bamako, Mali;; 2 Department of Oncology, North-Western University, Chicago, Illinois, United States;; 3 Department of Medicine, Pennsylvania State University College of Medicine, Hershey, Pennsylvania, United States;; 4 Department of Neurology, University of Chicago, Chicago, Illinois, United States

**Keywords:** Diet, Red meat, Gut microbiota, Colorectal Cancer, Dysbiosis

## Abstract

Excessive consumption of red and processed meat has been associated with a higher risk of developing colorectal cancer. There are many attempts to explain the risk of colorectal cancer associated with the consumption of red and processed meat:

- The temperature cooking of meat such as grilling and smoking contribute to the formation of mutagenic compounds including heterocyclic amines and polycyclic aromatic hydrocarbons.
- Heme iron in red meat is involved in the formation of N-nitroso compounds and lipid peroxidation products in the digestive tract.
- Fatty red meat is involved in the production of secondary bile acids by the bacteria of the gut microbiota.

Many of the products formed are genotoxic and can cause DNA damage and initiate carcinogenesis of colorectal cancer. Various mechanisms contributing to their genotoxic role have been established in human and animal studies. In addition, there is increasing evidence that compounds formed from red and processed meat interact with the gut microbiota in colorectal cancer pathways. Although several early studies in animals and humans suggest a direct causal role of the gut microbiota in the development of colorectal cancer, the links between diet, gut microbiota, and colonic carcinogenesis are largely associations rather than proven causal relationships. Various biological mechanisms, including inflammation and oxidative stress can lead to DNA damage, gut dysbiosis, and therefore increase the risk of colorectal cancer. Dysbiosis of the gut microbiota may increase the risk of colorectal cancer through dietary component promotion of colonic carcinogenesis. In this paper, we review and update current knowledge about the relationships between red meat consumption, gut microbiota, and colorectal cancer.

## INTRODUCTION

Colorectal cancer (CRC) is the third most common cancer, diagnosed in both men and women, with an estimated 1.9 million new cases and 935 000 deaths reported in 2020, worldwide [1–3]. However, substantial disparities in both CRC incidence and mortality exist by geographical region, as shown in Tables 1 and 2.

Rectal and colon cancers related deaths are estimated to be in 60% and 71.5% respectively in 2035), making CRC a worldwide public health concern [3–5]. Of major concern, as well, is the increasing incidence of cases and deaths in youth CRC pathophysiology is associated with multiple risk factors including diet, diabetes, obesity, lifestyle, genes, and specific diseases, such as Crohn’s disease, ulcerative colitis, and dysbiosis [6–8]. Epidemiological studies have highlighted specific diets that are likely to be associated with CRC risk. On particular, a red meat enriched diet, low fiber intake, and heavy alcohol intake have been shown to adversely affect the risk of CRC [9,10]. Because of the way they are preserved (combination of salt, nitrate or nitrite), processed meats are exposed to the formation of carcinogens during the high temperature cooking process [11,12]. Such a long-term regimen may promote an increased risk of CRC. Other modifiable risk factors, such as low levels of physical activity, being overweight and smoking may increase CRC risk [13,14]. This evidence suggests that the risk of CRC may be reduced by diet, in addition to health behaviors.

Of 478,040 participants enrolled in the European Prospective Investigation into Cancer and Nutrition (EPIC) trial who were prospectively followed between 1992–1998, 1,329 CRC cases were detected and an association with red and processed meat consumption was observed [15]. Recently, the International Agency for Research on Cancer (IARC) and World Cancer Research Fund-American Institute for Cancer Research (WCRF-AICR) concluded that there is sufficient evidence to support that high consumption of processed meat may increase CRC risk and that evidence of increased risk from red meat consumption is thought to be either putative or probable [16]. Indeed, excessive consumption of red meat has a significant impact on the composition and function of the gut microbiota [17–19]. The human digestive tract is home to no less than 1012 to 1014 microorganisms, including bacteria (the most abundant), viruses, parasites, and non-pathogenic fungi [20,21]. The gut microbiota contains10-fold more cells than the human body and about 150-fold more genes than the human genome [22]. The gut microbiota composition changes with diet, sex, age, race, and lifestyle [21]. After colonization at birth to about 2 years of age, the gut microbiota is unique to each individual before becoming stable, over time [20]. In addition, environmental pollutants such as heterocyclic amines (HCA), polycyclic aromatic hydrocarbons (PAH) that contaminate red meat during cooking process at high temperature and the metabolic by-products (N-nitroso compounds, secondary bile acids, heme, trimethylamine-N-oxide (TMAO)) may interact with the gut microbiota and promote CRC carcinogenesis [17,19,23–25]. Moreover, the microbiome community pattern of the gut is disrupted in patients with CRC compared to healthy individuals [26,27].

In this paper, we review and update current knowledge regarding the interaction between the gut microbiota and CRC, the impact of red meat consumption on the gut microbiota, and the contribution of red meat consumption to the pathogenesis of CRC.

## LITERATURE REVIEW

### Red meat consumption and colorectal cancer

**Figure 1:**
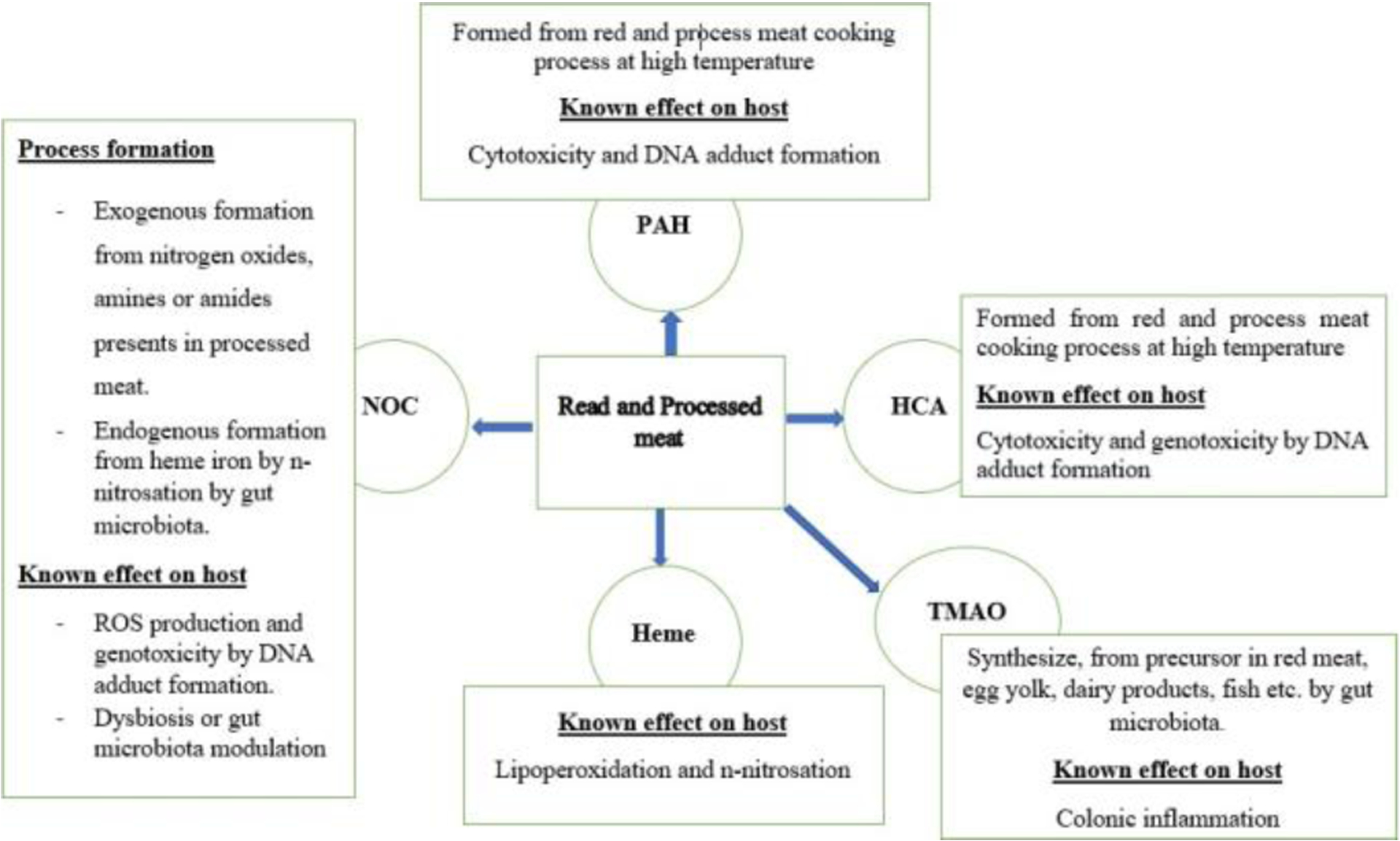
Read meat and processed increases CRC risk. Red and processed meat could promote colon carcinogenesis via different mechanisms such as toxic metabolites production, Lipoperoxidation and n-nitrosation, DNA adduct formation, and colonic inflammation. Red and processed meat can be contaminated by environmental pollutants, including HCA and PAH, during cooking. The International Agency for Research on Cancer (IARC) has classified the HCA and PAH as potential human carcinogens. The intestinal microbiota digests the proteins contained in ingested red meat to generate carcinogenic metabolites such as NOC, TMAO, and heme iron.

Numerous epidemiological and scientific studies suggest that the risk of CRC is increased by red and processed meat consumption [21,28,29]. According to WCRF/AICR reports published in 2007 and 2018, excessive consumption of red and processed meat convincingly increases the risk of CRC [30,31]. Red and processed meat was classified as a carcinogen at the same risk level as cigarettes and alcohol by the World Health Organizations International Agency for Research on Cancer in 2015 [32]. Because of its probable carcinogenicity, red and processed meat has been classified as a carcinogen 2A Group [33]. The risk of CRC was found to be increased by 16% for an additional 50 g/day of red and processed meat consumption and by 22% if consumption increased to 100 g/day [34]. Because of its nitrite conservation method, processed meat may present a higher risk per gram of intake than red meat [35,36]. Once produced, processed meat is preserved by methods other than freezing, including curing (the combination of salt, sugar and nitrate or nitrite), drying, smoking, cooking and packaging. According to traditional recipes specific to regions, this list of processed meat and conservation methods is not exhaustive, as there are many other manufacturing and conservation methods worldwide. However, these different conservation methods expose the meat to carcinogenic products (N-nitroso, HCA, HAP) formed during the cooking process at high temperature, especially brining and smoking [11,12]. To date, there are no clearly established biological mechanisms that could explain the role of red and processed meat in the process of CRC carcinogenesis. However, several hypotheses have been formulated and tested by experimental studies to try to explain how red and processed meat could increase the risk of CRC as shown in Figure 1. Experimentally the hypotheses that were tested are: (a) that high-fat meat might promote CRC carcinogenesis by induction of cytotoxic secondary bile acid production; (b) that high temperature meat cooking processes form mutagenic heterocyclic HCA and PAH; (c) that potentially carcinogenic N-nitroso compounds (NOC) are formed exogenously in meat and/or endogenously by nitrosation of amines and amides; (d) that heme iron from red meat may promote carcinogenesis through the formation of NOC and lipid peroxidation products.

**Figure 2:**
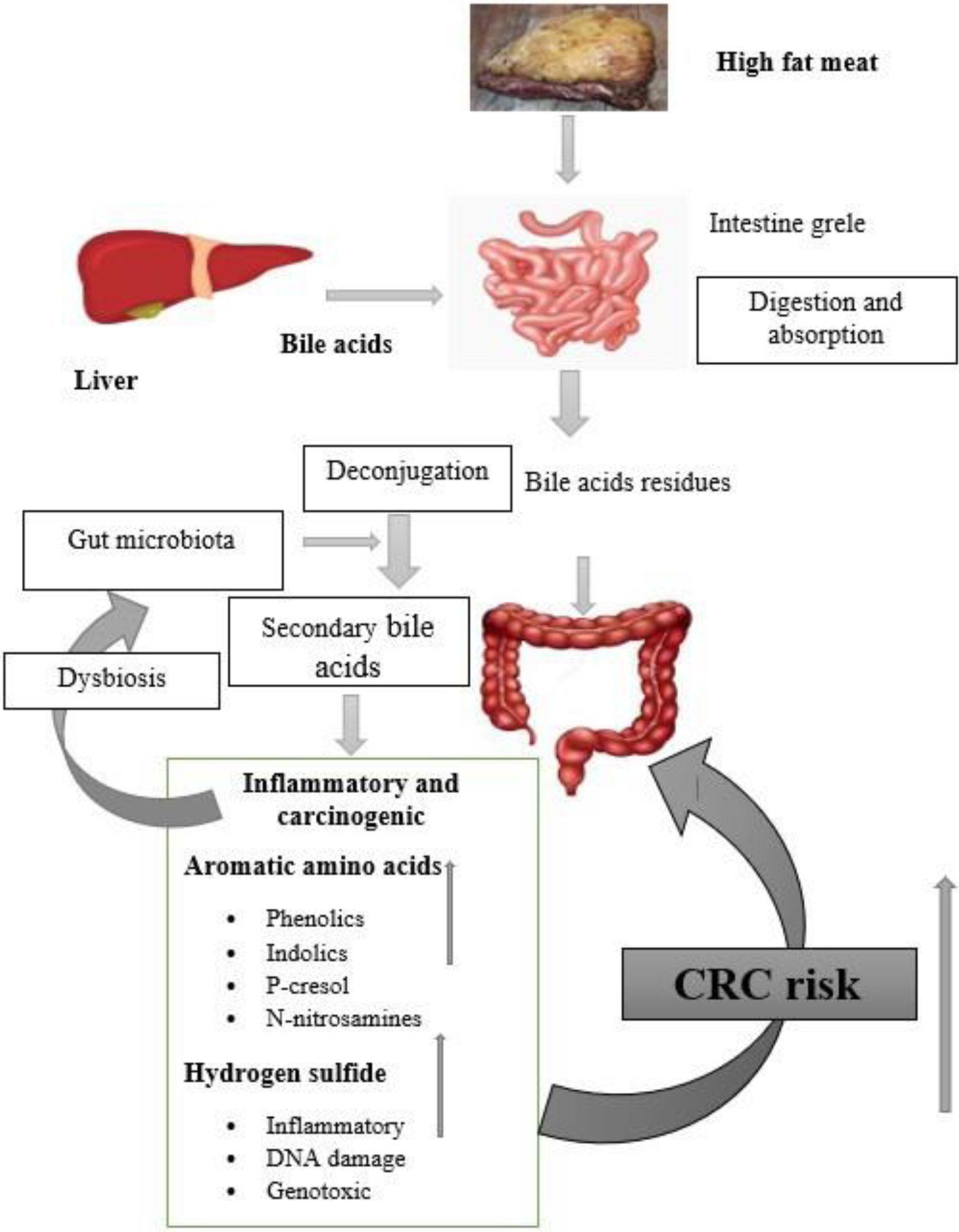
CRC risk related to the consumption of high-fat red meat. A high-fat diet upregulates the production of primary bile acid in the stomach. Some of this primary bile acid transits into the colon, transforming it into secondary BA by the gut microbiota. Secondary BAs are involved in several mechanisms leading to CRC carcinogenesis, including producing genotoxic substances (aromatic amino acids, hydrogen sulfide) and dysfunctional gut microbiota metabolism.

Ingestion of red meat rich in fat and excessive secretion bile acid (BA) have been cited in several animal and human experimental studies as one of the factors promoting the increased risk of CRC. Primary BAs, including cholic acid (CA) and chenodeoxycholic acid (CDCA), are synthesized by the liver following the digestion of dietary lipids in the stomach to emulsify fats. After lipid digestion, most primary BAs are deconjugated and reabsorbed (enterohepatic recycling). Still, a small amount may pass into the colon where they are transformed into secondary BAs (deoxycholic acid and lithocholic acid) by colonic bacteria by enzyme 7α-dehydroxylation as shown in Figure 2. The genus Clostridium is the main 7α-dehydroxylation-producing colonic bacterium [22,26,37]. Mice fed with deoxycholic acid developed significant intestinal inflammation 24 weeks after accumulation of deoxycholic acid in their feces [38]. A 3 to 4-fold higher level of secondary BA has been reported in a study of African-Americans who consumed a high-fat diet compared to native Africans who consumed a low-fat diet, suggesting an elevated risk of CRC in African Americans [39]. A similar study, in 2015, based on a change in the diet of African-Americans, from a high-fat to a low-fat diet, showed a significant decrease in secondary BA secretion and a decrease inflammation of the colonic mucosa [40]. BA with detergent properties can damage the colonic epithelium when presented in high concentrations in the colon. This destruction of colonic cells can lead to inflammation and increased proliferation of stem cells in the colon, leading to a pre-cancerous state [41]. Secondary BA may also promote the growth and multiplication of transformed stem cells in the colon by modulating the Wnt/β-catenin and M3R signaling pathways in cancer cells [42]. Short-term exposure to secondary BAs induce reactive oxygen species (ROS) production in the colon responsible for DNA damage. In contrast, long-term exposure causes inhibition of the tumor suppressor gene (TP53) or activation of the PI3K/Akt signaling pathway in colonic cells contributing to CRC carcinogenesis [43–46]. Through the farnesoid X receptor located on the nuclear membrane of colon cells, deoxycholic acid can mediate CRC carcinogenesis by inhibiting mucosal scarring in an in vivo mouse model [47].

HCA are formed from products in processed meat (creatinine, amino acids, sugars) when cooked at high temperature [48]. The formation of HCA depends on the type of meat, temperature, time and cooking method. In addition to red and processed meat, poultry and fish also have HCA. The greatest amounts of HCA are produced during high temperature cooking processes such as grilling, frying and barbecuing [49]. The main HCA formed are 2-amino-3,4,8-dimethylimidazo (4,5-f) quinoxaline (DiMeIQx), amino-3-methylimidazo (4,5-f) quinolone (IQ); 2-amino-1-methyl-6-phenylimidazo(4,5-b) pyridine (PhIP); 2-amino-3,8-dimethylimidazo(4,5-f) quinoxaline (MeIQx); and 2-amino-3,4-dimethylimidazo (4,5-f) quinoline (MeIQ). The International Agency for Research on Cancer (IARC) has classified the HCA PhIP, MeIQ, and MeIQx as potential human carcinogens, while IQ is considered a probable human carcinogen [33,50]. In 2015, a Japanese study, of colonoscopies of Japanese women, showed that a high concentration of MeIQ with exposure to PhIP was correlated with a high risk of CRC [51]. A European prospective cohort study of 25,540 participants similarly reported that PhIP was associated with a high risk of colorectal adenoma [52]. Conversely, several other studies have shown that an increase in the co-occurring concentration of the HCA DiMeIQx, MeIQx, and PhIP are positively associated with a significant risk of colorectal adenoma [49,53]. HCA are genotoxic and carcinogenic in animal models. They are involved in the formation of DNA adducts following an N-oxidation reaction catalyzed by the cytochrome P450 enzyme and an O-esterification by N-acetyltransferases [54,55]. PAH are formed from high-temperature domestic cooking and industrial activities as a result of the incompetent combustion of organic materials (coal, crude oil, and gasoline). During cooking, especially red and processed meat, food may be contaminated with PAH over an open flame. With the same action characteristics as HCA, PAH may also increase the risk of CRC in humans. (49) PAH, mainly benzopyrenes or called benzo(α)pyrene (B(α)P), have been suspected to increase the risk of CRC in humans [49]. In experimental studies, particularly in mice, B(α)P were implicated in different mechanisms of CRC carcinogenesis, including DNA adduct formation, induction of oxidative stress, and increased expression of proinflammatory cytokines with dysregulation of the wnt/β-catenin signaling pathway [56,57].

Hughes et al. reported, for the first time in 2001, a significant excretion of NOC in the feces of volunteers who consumed large amounts of red meat [58]. NOC are formed exogenously from nitrogen oxides and amines or amides present in processed meat on the one hand and on the other hand endogenously by decarboxylation of the gut microbiota, followed by n-nitrosation in presence of nitrite [15,49]. These are most often found in certain processed foods such as smoked cheese, smoked fish, cold cuts, ham, sausages. It has been shown that heme iron lead to increased endogenous formation of NOC including N-nitrosothiols, and nitrosylated heme in the gastrointestinal tract during digestion of red meat [59]. These NOC formed can alkylate guanine at the O6 position on DNA resulting in the formation of promutagenic O 6-methylguanine and O 6-carboxymethylguanine lesions, which if not repaired quickly by the DNA repair enzyme O 6-methylguanine-DNA methyltransferase, could lead to genetic mutations and subsequently to the development of CRC [60–62]. Furthermore, in another study, a group of DNA adducts related to NOC and lipid peroxidation were identified as potential markers of red meat digestion. These DNA adducts include methylguanine; 3,N4-etheno-C; guanidinohydantoin; carboxyethyl-T; dimethyl-T; hydroxymethyl-T; tetramethyl-T; 6-carboxymethylguanine; and hydroxyethyl-T [63]. In addition, DNA adducts related to nitroso compounds and lipid peroxidation have been implicated in multiple genetic alterations by causing mutations in key colon cancer genes such as Adenomatous Polyposis Coli (APC), tumor suppressor gene (TP53) and Kirsten rat sarcoma virus (KRAS) [64].

This evidence suggests that NOC, formed from red and processed meat, can promote the development of CRC by inducing mutations in key tumor suppressor genes, including APC and TP53, and oncogenes such as KRAS.

The carcinogenic role of heme iron in red and processed meats is supported by numerous epidemiological and experimental studies [64,65]. Three main mechanisms of heme-induced colorectal carcinogenesis have been described: cytotoxicity of heme by accelerating programmed cell death and epithelial hyperplasia; heme-induced lipid peroxidation and DNA adduct formation and mutation of the APC gene; and heme catalysis of NOC resulting in genetic mutation as explained above [66–69]. Heme is catalyzed by transforming hydroperoxides into ROS [64]. The ROS formed have been suspected of being responsible for the cytotoxic effect of heme, according to a study by Pierre et al. in 2007 [70]. An in vitro study, performed on colonic cells, showed that a heme concentration higher than 100 μM resulted in colonocyte toxicity [71]. Heme increases the membrane permeability of the colonocytes, which subsequently leads to cell lysis.(64) Similarly, in 2012, Ijessennagger et al. showed that mice fed a high heme concentration during the first two days old exhibited acute oxidative stress of their colonocytes, as revealed by the release of Vnn1, a marker of oxidative stress. Cross et al., in 2003, in an evaluation of the effect of heme iron supplementation of a red meat diet, showed a significant increase in the fecal concentration of NOC [72]. Similarly, in 2010, they found a strong correlation between CRC risk and consumption of heme iron, nitrates, and processed meat [73].

The mechanism of trimethylamine–N-oxide (TMAO)-mediated CRC carcinogenesis in humans and animals has been examined in several studies [74]. TMAO is a metabolite of the gut microbiota produced from precursor molecules (choline, L-carnitine, phosphatidylcholine, betaine), which are abundantly present in red meat, egg yolk, dairy products, fish, vegetables, and fruits. The precursors of TMAO are converted to trimethylamine by the gut microbiota, then absorbed in the small intestine, and transported by the portal vein to the liver where they interact with flavin monooxygenase 3 (FMO3) and produce TMAO [26,75].

The inescapable role of the gut microbiota in the generation of TMAO was demonstrated by a study of antibiotics given to human subjects for one week to eliminate the gut microbiota [76]. The elimination of the gut microbiota by antibiotics was positively associated with decreased plasma and urinary TMAO levels compared to untreated controls. The same phenomenon has been reported in animal experiments [77]. In 2014, a study showed a correlation between plasma TMAO levels and CRC in women with low plasma vitamin B12 levels [78]. A similar study, in 2017, revealed that the precursor choline was associated with a 3-fold increased risk of CRC in men with elevated blood levels [79]. Evidence has suggested that TMAO may promote inflammation via various mechanisms, including increased expression of pro-inflammatory genes (cytokine genes, IL6, TNF-α, and chemokine ligands CXCL1, CXCL2) and increased pro-inflammatory effects mediated by *H. pylori* infection in the stomach [80–84]. The generation of N-nitroso compounds, known as genotoxic agents, is another mechanism of TMAO involved in the carcinogenesis of CRC [85]. It may also be involved in NLRP3 inflammasome activation and ROS production in human colonic cells, preventing their down-regulation [86]. In view of this evidence, CRC carcinogenesis may be activated by TMAO pro-inflammatory roles.

### Red meat consumption and gut microbiota

The interaction between compounds formed from red meat directly or indirectly after its ingestion, including N-nitroso compounds (NOC), heterocyclic amines (HCA), polycyclic aromatic hydrocarbons (PAH), heme iron, bile acids (BA), Trimethylamine-N-oxide (TMAO) and the gut microbiota has been reported in numerous animal and human studies as shown in Table 3. Naturally, the number of NOC-producing bacteria (*Escherichia*, *Pseudomonas*, *Proteus*, *Klebsiella*, and *Neisseria*) is low in humans, but may increase with a diet rich in nitrates or nitrites [32,87–89]. Nitrates are found in high concentrations in processed meats and in certain foods, such as vegetables (beets, celery, lettuce, radishes, spinach) [90]. Nitrate ingested through these foods is reduced to nitrite by oral and digestive tract bacteria. The nitrites once formed react with amines, amides and other precursors by nitrosation in the gastrointestinal tract to form NOC compounds. Animal studies have shown that NOC compounds are potent carcinogens in animals [91–93]. In mice, for example, the colon in mice with colitis is enriched with *E. coli* during an intestinal inflammatory response mediated by nitrate compounds [17]. Previous studies have not been able to provide consistent evidence of an association between exposure to NOC and an increased risk of CRC in humans [94,95]. A study of European populations showed an association between N-nitrosodimethylamine from food sources and increased risk of CRC [94]. Although there have been few studies of NOC compounds and increased risk of CRC, there is strong evidence of an association between red and processed meat consumption and increased risk of CRC [28–32]. Therefore, excessive consumption of processed meat can lead to intestinal dysbiosis and a high risk of CRC carcinogenesis by promoting the multiplication of NOC-producing bacteria [26,96,97].

It is not known, however, whether or not the combined action of nitrate-induced *E. coli* multiplication and the formation of NOCs are responsible for promoting inflammation or the development of CRC. Exposure of mouse models to PAHs also causes change in the composition of the gut microbiota after colonic inflammation. In humans, PAHs and HCAs altered the volatile profile of the fecal microbiota and their metabolite activities after high exposure of the gut microbiota over 24 hours to these substances [23,98,99].

Certain bacteria may reduce the risk of CRC associated with HCA consumption in the gut microbiota (e.g., *Eulonchus halli*) through their beta-glucuronidase and glycerol/diol dehydratase activities that convert HCA to HCA-M1 [99,100].

The production of TMAO from precursors such as choline, L-carnitine, phosphatidylcholine, and betaine is influenced by the gut microbiota in humans [24]. TMAO precursors are biodegraded by gut microbiota to generate pro-inflammatory molecules. The Eubacterium limosum has proved to be very effective in converting TMAO precursors by demethylation of L-carnitine and reducing the amount of TMAO produced in the gut [101]. Mice receiving a diet rich in heme increased the number of bacteria such as Proteobacteria or Bacteroidetes accompanied by a reduction of Firmicutes bacteria and Deferribacteres in their intestine [18,19,102].

Other infectious agents, including viruses bovine origin, thermoresistant, and potentially oncogenic, have also been reported as agents that may be involved in the process of CRC carcinogenesis [103]. Nowadays, only the transmission of heat-resistant bovine viruses during the meat consumption is suspected as an infectious factor involved in the mechanisms of CRC carcinogenesis [104]. These are mainly polyomaviruses, papillomaviruses, and probably the Torque Teno virus (TTV). Once consumed through beef contaminated by environmental substances or by substances generated during the cooking of the meat (HCA, PAH) these viruses could initiate CRC by synergistic interaction with these substances [103]. However, no experimental evidence has shown a direct correlation of these infectious agents with CRC development. This new hypothesis deserves further attention and exploration.

Based on this evidence, the gut microbiota and compounds derived from red meat appear to have close interactions that may influence the composition of the gut microbiota leading to protection against CRC or exposure to CRC risk.

### Interaction between gut microbiota and colorectal cancer

In 1975, for the first time, the link between gut microbiota and CRC was established in germ-free compared to conventional rats, with the development of a colorectal tumor after chemical induction [105]. They found that 93% of the conventional rats developed multiple colon tumors and only 20% of the germ-free rats developed colon tumors. After subcutaneous injection of azoxymethane in both groups of rats, the incidence and multiplicity of colonic tumors were increased in germ-free rats compared to conventional rats [106]. Gut microbial dysbiosis in mice was observed during both spontaneous and chemically induced colon tumorigenesis. The diversity of the gut microbiota was reduced in C57BL/6J Apc Min/+ mice compared with wild-type C57BL/6J mice [107]. Since then, several studies found disruption of some microorganisms in the gut microbiota of patients with CRC compared to healthy controls [108]. CRC patients showed a reduction in bacterial diversity and richness compared to healthy individuals [109].

Whole genome sequencing of gut microbial species has allowed researchers to study the microbial communities that colonize colonic tumors as well as non-tumor colonic sites and to characterize individual oncogenic microbiomes [110]. The proliferation of certain bacterial populations including *Helicobacter pylori* (*H. pylori*), *Escherichia coli* (E.coli), *Streptococcus bovis* (*S. bovis*), *Enterococcus faecalis* (*E. faecalis*), *Clostridium septicum* (*C. septicum*), *Fusobacterium nucleatum* (*F. nucleatum*), Enterotoxigenic Bacteroides fragilis (ETBF) and *Streptococcus gallolyticus* (*S. gallolyticus*) were suspected as factors promoting CRC [108,111]. To better understand the role of the gut microbiota in the carcinogenesis and progression of CRC, hypotheses have been proposed. For example, the driver–passenger model has been proposed to classify commensal bacteria into two different groups, the driver bacteria and the passenger bacteria [112]. Driver bacteria cause DNA damage in colonic cells, that can initiate or cause CRC progression in the first spatial location, then the tumor microenvironment changes to promote infiltration and proliferation of passenger bacteria that may be dominant later in the colonic tumor site. This model attempts to explain that the driver bacteria in initiating CRC, will not always exist as an oncogenic marker in the tumor environment, but will disappear and will be replaced by passenger bacteria in the cancerous tissue. This model can help to clearly understand the discrepancy between results in different studies, clarifying the ambiguous relationship between gut microbiota and CRC.

The other model proposed is the keystone model, which supports the role of a key pathogen in the process of dysbiosis associated with a given disease [113]. This hypothesis is not based on the abundance or level of strength of the microbiota related to the disease, but on its functions that contribute to dysbiosis and its maintenance. For example, *Klebsiella pneumonia* and *Proteus* mirabilis could be treated as key pathogens of inflammatory bowel disease, and the role of ETBF in CRC. This model may provide new insights to review the potential role of gut pathogens in the initiation and progression of related disorders.

The potential role of *F nucleatum* in CRC carcinogenesis has been reported through studies [114]. Analysis of rectal mucosa, feces, and tumor samples from CRC patients showed a high prevalence of bacteria belonging to the genus Fusobacterium compared to healthy subjects or remotely adjacent healthy mucosa in these same CRC patients [115]. *F. nucleatum* infiltrates the colonic tumor through its adhesin (FadA), selectively binding to E-cadherin and activates the β-catenin signaling pathway, inducing inflammatory responses allowing CRC progression [116]. *F. nucleatum* also inhibits T cell and natural killer cell activity through another adhesin, fibroblast activation protein 2 (Fap2), which binds to T cell immunoglobulin and the ITIM domain [117]. Fap2 adhesin is used by *F. nucleatum* to infiltrate the colonic tumor by binding to the carbohydrate moiety D-galactose-β (1–3)-N-acetyl-D-galactosamine (Gal-GalNAc), which is overexpressed in CRC cells [118]. Interleukin IL-17A is highly expressed in CRC patients with *F. nucleatum*-enriched colonic tumors [119]. A recent study showed that CRC cell metastasis was dependent on the *F. nucleatum* adhesin Fap2, which induced the secretion of the pro-inflammatory cytokines, IL-8 and CXCL1 [120]. The carcinogenic property of *F. nucleatum* in Apc Min/+ mice and in human CRC cell lines was indicated by Nuclear factor-kappa B (NF-κB) activation which in turn induced miR21 gene expression promoting inflammatory responses [121]. Another recent study showed that *F. nucleatum* was significantly increased in patients with early stage CRC and that the presence of *F. nucleatum* in CRC tissues is associated with a poor prognosis of the disease [122,123,124].

Several studies have reported the link between ETBF and CRC and its use as a potential biomarker in the diagnosis of CRC [125–127]. ETBF secretes a 21 kDa *B. fragilis* toxin (BFT) that cleaves E-cadherin on colonic epithelial cells resulting in disruption of the colonic barrier [128]. The disrupted colonic barrier causes diarrhea and inflammatory bowel disease [129,130]. A recent study reported that *E. faecalis* and ETBF copy number were significantly higher in CRC tissue samples compared to the no CRC group [130]. Infection of Apc Min/+ mice with ETBF induced selective activation of Signal transducer and activator of transcription 3 (STAT3) with CRC characterized by Th17 responses. ETBF promotes CRC progression by secreting particles that stimulate colonic epithelial cells to produce exosome-like nanoparticles containing high levels of sphingosine-1-phosphate, CCL20, and prostaglandin E2 (PGE2) that are required for recruitment of Th17 cells into CRC tissues to support their growth and survival [131]. ETBF also plays an important role in mediating inflammatory responses during CRC carcinogenesis and chronic inflammatory bowel disease by activating the NF-κB signaling pathway to recruit immature polymorphonuclear myeloid cells [132,133]. ETBF promoted inflammation and CRC cells multiplication by downregulating exosomal miR-149–3p both in vitro and in vivo [134].

The pilus 3 (pil3) of *S. gallolyticus* is essential for its attachment to human mucus-producing epithelial cells (135). Interestingly, pil3 binds both to the human mucin 2 (MUC2), which predominates in healthy colonic tissue, and to mucin 5AC (MUC5AC), which is overexpressed in cancerous colonic tissue [135]. It has been postulated that commensal colonization of *S. gallolyticus* is facilitated by its binding to the mucin MUC2, while it’s binding to MUC5AC gives a growth advantage to bacterial species of the gut microbiota in the tumor microenvironment [136]. This helps explain the higher carriage rate of *S. gallolyticus* in the presence of colonic tumors. Colonic epithelial cells from CRC patients showed high NF-κB gene expression in the S. gallolyticus-positive group compared with the S. gallolyticus-negative group [137]. NF-κB plays an important role in mediating inflammatory responses during chronic inflammatory bowel disease and CRC carcinogenesis. In another study the same authors showed strong expression of IL-1 and Cyclo-oxygenase-2 (COX-2) genes by colonic cells of *S. gallolyticus* seropositive CRC patients, both of which are products of NF-κB activity [138].

*E. coli*, is an anaerobic gram-negative commensal bacterium, commonly found in the intestinal microenvironment. Some groups of *E. coli* called pathotypes belonging mainly to the B2 and D phylogroups have been identified as potentially oncogenic and pro-inflammatory [139]. Several studies have linked these pathogenic *E. coli* groups to CRC risk. Enrichment in B2 and D phylogroups was reported in CRC patients compared to control subjects (without CRC) [140]. Indeed, these phylogroups were identified in 90% and 93% of adenoma and carcinoma patients, respectively, while only 3% of colon biopsies from asymptomatic controls were positive for these phylogroups. A similar study showed that among 21 CRC patients 70% had *E. coli* enriched colorectal tissue, compared with 42% of 24 control biopsies without CRC [141]. In addition, many other studies have confirmed the enrichment of tumor tissues with *E. coli* in CRC patients compared to healthy subjects [142–144]. Therefore, a link between these pathogenic *E. coli* groups and CRC risk has been proposed through several studies. Nevertheless, the mechanism involved is very poorly understood to date. But according to studies, pathogenic *E. coli* strains producing cyclomodulins, and toxins are responsible for induction of DNA damage and cell cycle disruption in eukaryotes [131,139,145]. A high prevalence of cyclomodulin and toxin-producing *E. coli* was observed in CRC patients compared to healthy subjects [139,143]. These genotoxic toxins include the polyketide synthase (pks) pathogenicity responsible for colibactin expression, cytolethal distention toxin (CDT), cytotoxic necrotizing factor (CNF), cycle-inhibiting factor (CIF), and afimbriale adhesin (afa) [144,146,147]. The pks genomic island codes for the polyketide-peptide genotoxin, colibactin [148–150]. Culture of mammalian epithelial cells exposed to pks+ *E. coli* to show transient DNA damage [149]. According to experimental studies, colibactin could promote tumor growth by forming cross-links with DNA in cellulo as an alkylating agent, and DNA double-strand breaks or by promoting the emergence of senescent cells with an irreversible cell cycle arrest [151,152]. In AOM/IL10−/− or ApcMin/+ mouse models with chemo-induced tumor, infection with colibactin-producing *E. coli* strains induced an acceleration of tumor development compared to uninfected control mice or mice infected with a non-colibactin-producing mutant of these strains, or animals infected and treated with molecules inhibiting colibactin synthesis [139,142,144,153]. CNF toxin binds to the tight junctions of colonic epithelial cells which internalize it by endocytosis and promotes cell proliferation by encouraging entry into the cell cycle and the G1/S transition for cell survival by inducing, in particular, the expression of the anti-apoptotic proteins Bcl-2 (B-cell lymphoma 2) and Bcl-xl (B-cell lymphoma-extra-large) [154]. CIF promotes actin cytoskeleton rearrangement and induces G2/M cell cycle arrest characterized by inactive phosphorylation of cyclin-1-dependent kinase, an essential player in cell cycle regulation [155]. CNF-1 also induces transient activation of COX-2 and Rho GTPases characterized by alterations in the cytoskeleton and thus affects the cell cycle [156,157]. The CDT toxin secreted by pathogenic *E. coli* strains is known to have DNAase activity resulting in DNA double strand breaks, cell division arrest and inhibition of cell apoptosis [157].

Like *E. coli*, *E. faecalis* is part of the facultative anaerobic Gram-positive commensal flora and does not appear to be offensive to humans. However, studies have shown enrichment of CRC patients’ fecal samples and tumor tissues by *E. faecalis* compared to those healthy individuals [158,159]. A recent study found that *E. faecalis* species was significantly lower in obese patients than in non-obese patients with CRC [160]. Similarly, the abundance of *E. faecalis* was relatively higher in obese subjects than in non-obese subjects. This study demonstrated that a reduced presence of *E. faecalis* may be associated with obesity-related CRC carcinogenesis. Another recent study conducted on 256 fresh frozen CRC tissues detected *E. faecalis* in 193 of the 256 CRC tissues [161]. *E. faecalis* bacteremia was observed in an 86-year-old white male during a secondary gastrointestinal hemorrhage with confirmation of colorectal adenocarcinoma by colonoscopy [162]. *E. faecalis* was able to promote and maintain colitis in Il10−/− or Il10 gene deficient mice with induction of rectal dysplasia and carcinoma [163]. Intestinal epithelial cells from wild-type mice expressed the cytokine TGF-β upon infection with colitogenic *E. faecalis*, thereby activating Smad signaling. Interestingly, these mice lost toll-like receptor (TLR2) expression with NF-κB-dependent pro-inflammatory gene inhibition, in contrast to Il10−/− mice that failed to inhibit TLR2 receptor-mediated pro-inflammatory gene expression in intestinal epithelial cells upon colonization by *E. faecalis*. (163) Blood isolates of *E. faecalis* have also been associated with the production of extracellular superoxide (O2−) and hydrogen peroxide (H2O2) in the intestine [164,165]. These extracellular free radicals induced DNA damage in the studies [165]. Similarly, *E. faecalis* is able to induce DNA damage in vivo in colonic cells in rats. Extracellular infection of mammalian cells by *E. faecalis* can result in the production of superoxide (O2−) leading to overexpression of COX-2 in macrophages and promotes chromosomal instability in primary colonic epithelial cells [166]. Similarly *E. faecalis* is able to polarize colonic macrophages into an M1 phenotype, which in turn induces aneuploidy and chromosomal instability in colonic epithelial cells commonly found in cancers [167]. These data may explain the mechanisms by which *E. faecalis* exerts its impact on CRC.

McCOY WC et al. first established the link between *S. bovis* and CRC in 1951 [168]. Subsequently, around 1977, the *S. bovis* strain was isolated from stool samples of 35 out of 63 CRC patients compared to 11 out of 105 control individuals without CRC [169]. Subsequent studies have confirmed the link between *S. bovis* and CRC [170,171]. According to an in vitro study carried out in 2004, infection of colonic cells by *S. bovis* induced an overexpression of pro-inflammatory mediators, notably IL-8, COX-2 and PGE2 [172]. Other animal studies haveconfirmedthecarcinogenicpropertiesof*S.bovis*.Azoxymethane-treated rats confirmed the release of pro-inflammatory mediators after infection with *S. bovis*, which explains the increase in the number of aberrant crypts. Interestingly of the azoxymethane-treated rats, three of the six treated rats developed polyps during *S. bovis* infection, whereas no polyps were detected in the uninfected azoxymethane treated rats. Hyperproliferative crypts were also detected in azoxymethane-treated rats with *S. bovis* infection, demonstrating the involvement of this bacterium in CRC carcinogenesis [173]. Through samples of human origin including stool, tumor and non-tumor CRC tissues, studies have shown an enrichment of this bacterium in these samples in CRC patients compared to control subjects without CRC [138]. Studies have also reported significant overexpression of mRNAs that encode the proinflammatory mediators IL-1β, COX-2, and IL-8 in *S. bovis*-infiltrated tissues compared with non-infiltrated tissues. Similarly the expression of these mRNAs was elevated in tumor tissues compared to non-tumor tissues. The pro-inflammatory profile of *S. bovis* may increase the risk of CRC development and progression.

*H. pylori* is a gram-negative bacterium that preferentially infects and colonizes gastric tissue in humans. Although most infected individuals remain asymptomatic, *H. pylori* can induce chronic inflammatory responses increasing the risk of gastric ulcer, and adenocarcinoma of the stomach [174]. Despite gastric colonization by *H. pylori*, it has been shown that its toxicity can extend outside the stomach. The link between *H. pylori* infection and CRC remains controversial with studies showing a strong association with a high prevalence of *H. pylori* infection in patients with colonic adenomas and carcinomas [175,176]. While other studies have shown no association [177,178]. Many recent studies have reported a significant association between *H. pylori* infection and an increased occurrence of CRC [179–181]. Similarly Yan et al. showed a positive association between *H. pylori* infection and CRC. Despite the ambiguity between some studies on the link between *H. pylori* infection and CRC, others have attempted to explain the molecular mechanism underlying the potential association between *H. pylori* infection and CRC with some supporting hypotheses such as toxin release, dysbiosis and chronic inflammation. For example, significant gastrin secretion mediated by *H. pylori* infection was associated with increased expression of COX-2 and anti-apoptotic B-cell lymphoma 2 (BCL2) protein compared to pro-apoptotic BCL2 Associated X(BAX) protein resulting in decreased apoptosis in CRC [182]. High gastrin production disrupts gastric acid production and the gastric barrier, leading to an imbalance in the intestinal microbiota [183]. This dysbiosis could facilitate the colonization and multiplication of oncogenic bacteria associated with CRC such as *B. fragilis* and *E. faecalis*. Other mechanisms of CRC carcinogenesis mediated by *H. pylori* infection have been proposed. These include the production of ROS and reactive nitrogen species (RNS) that can lead to DNA damage, which could promote CRC carcinogenesis [184]. In addition, *H. pylori* strains toxicities’ vary from patients. For example, strains that carry the virulence factor cytotoxin-associated gene A (CagA) are more toxic than those without, and patients with these strains have an increased risk of developing gastric cancer and CRC compared to those without [185]. The VacA protein secreted by these *H. pylori* strains can induce production of pro-inflammatory mediators such as TNF-α, IFN-γ, IL-1β, IL-6 and IL-8 by infected cells [186,187]. The toxins secreted by *H. pylori* give it a pro-inflammatory property that can promote gastric and CRC carcinogenesis.

*C. septicum* is a Gram-positive, anaerobic, spore-forming bacillus, which is not normally present in the intestinal flora, but can cause direct and spontaneous infections in the gut.

This bacterium produces alpha-toxin, a virulence factor which is both lethal and hemolytic in mice [188]. Several studies have reported a probable association between *C. septicum* infection and CRC [189–191]. This association may be due to the hypoxic and acidic tumor environment that favors germination of *C. septicum* spores [190]. Eighty percent (80%) of patients infected with *C. septicum* were associated with malignancy. The alpha-toxin-producing *C. septicum* group is associated with the release of TNF-α after activation of the mitogen-activated protein kinase (MAPK) pathway, which has been shown to be dysregulated in cancers [192–201]. This pro-inflammatory property of *C. septicum* may promote carcinogenesis. However, despite available data, no direct link between *C. septicum* and CRC has been defined to date.

## DISCUSSION AND CONCLUSION

This review examined current knowledge about risk factors of CRC carcinogenesis. Red and processed meat consumption and its interaction with the gut microbiota are found to be major associated factors. The CRC-associated gut microbiota is made of pro-inflammatory or pro-carcinogenic bacteria and opportunistic pathogenic bacteria that enrich the tumor microenvironment by promoting disease progression. Bacteria such as *E. coli*, S. gallolyticus, and *F. nucleatum* are frequently initiators of colonic carcinogenesis through virulence factors and responsible for CRC progression after their infiltration into the tumor microenvironment. Animal and human experimental studies also strongly support the evidence of diet being a major risk factor for CRC. More longitudinal clinical studies are needed to confirm and better understand the mechanisms underlying the diet-mediated disruption of gut microbiota in humans and establish the direct cause and impact that dysbiosis has on the initiation and progression of CRC.

## Figures and Tables

**Table 1:** Region-specific incidence rates by sex for cancers of the colon and rectum in 2020.

Region	Males/ Incidence rate per 100,000 residents	Females/ Incidence rate per 100,000 residents	Males/Incidence rate per 100,000 residents	Females/Incidence rate per 100,000 residents
	Colon cancer	Colon cancer	Rectum cancer	Rectum cancer
Southern Europe	25.3	16.4	14.1	7.3
Northern Europe	23.2	18.8	15.1	8.4
Australia/New Zealand	22.8	20.0	13.6	7.7
Eastern Europe	21.1	14.0	16.9	8.9
Western Europe	20.0	15.1	13.3	6.8
Northern America	17.4	15.0	11.0	6.6
Eastern Asia	16.4	13.0	14.6	7.7
Caribbean	14.1	13.8	3.9	3.4
South America	12.8	10.8	7.2	5.0
Micronesia/Polynesia	12.3	7.9	7.0	5.0
Western Asia	11.7	8.7	7.9	5.2
South-Eastern Asia	9.5	6.3	8.5	5.3
Central America	8.7	7.0	3.0	1.9
Southern Africa	8.7	5.9	7.2	5.0
Melanesia	6.7	3.4	5.8	3.9
Northern Africa	5.9	5.4	4.1	3.4
Eastern Africa	4.2	3.5	3.5	3.1
Western Africa	4.0	2.9	3.0	2.2
South-Central Asia	3.4	2.2	2.8	1.9
Middle Africa	2.9	2.3	3.6	2.8

**Table 2:** Colorectal cancer new cases and deaths by geographic region (2020).

Region	New cases	Deaths
Asia	957,896 (51.8%)	461,422 (52.4%)
Europe	499,667 (27.0%)	242,483 (27.5%)
North America	179,715 (9.7%)	64,105 (7.3%)
Latin America, Caribbean	128,006 (6.9%)	64,666 (7.3%)
Africa	61,846 (3.3%)	40,034 (4.5%)
Oceania	22,332 (1.2%)	8,066 (0.9%)

**Table 3:** Interactions between red meat-associated agents and gut microbiota that may increase CRC risk.

Food ingredients	Derived compounds	Action of derived compounds on gut microbiota	Action of gut microbiota on derived compounds	References
	NOC	Promotes selective growth of NOC-producing bacteria, creating a state of dysbiosis that is the origin of CRC.	-	(89)
	HCA and PAH	Promote colonic inflammation by altering the abundance, composition and metabolic activities of the gut microbiota.	Transform HCA and PAH into less toxic substances.	(23,99,100,193–195)
Read and processed meat	TMAO	-	Involved in the synthesis of TMAO from precursors such as choline, L-carnitine, phosphatidylcholine, and betaine.	(76,196–198)
	Heme	Increases proteobacteria and Bacteroides abundance; reduces Firmicutes and Deferribacteres; promotes adenoma formation; decreases stool butyrate levels.	Increased heme-induced lipoperoxidation, hyperproliferation ofcolonic tissue.	(18,19,102,199)
Fats	Secondary BA	Increases the ratio of Firmicutes/Bacteroidetes, which is associated with obesity; also promotes the increase of mucin-degrading Actinobacteria; reduces the abundance of Bifidobacterium, Lactobacillus and Akkermansia considered as the good bacteria of the gut microbiota.	Transforms primary bile acids into secondary bile acids which are involved in inflammatory bowel diseases.	(26,27,44,200,201)

**Note:** HCA: heterocyclic amines, PAH: polycyclic aromatic hydrocarbons NOC: N-nitroso compounds, Secondary BA: secondary bile acids, TMAO: trimethylamine-N-oxide

## ABBREVIATIONS

AFA: Afimbriale adhesin
APC: Adenomatous Polyposis Coli
AKT: Protein Kinase B
BA: Bile Acid
B(α)P: Benzo(Α)Pyrene
BAX: BCL2 Associated X
BCL-2: B-Cell Lymphoma 2
Bcl-xl: B-Cell Lymphoma- Extra-Large
CA: Cholic Acid
CagA: Cytotoxin-Associated Gene A
CDCA: Chenodeoxycholic Acid
CDT: Cytolethal Distention Toxin
CIF: Cycle-Inhibiting Factor
CNF: Cytotoxic Necrotizing Factor
CXCL1: Chemokine (C-X-C Motif) Ligand 1
CXCL2: Chemokine (C-X-C Motif) Ligand 2
CCL20: C-C Motif Chemokine Ligand 20
COX-2: Cyclo-Oxygenase-2
CRC: Colorectal Cancer
DiMeIQx: 2-Amino-3,4,8-Dimethylimidazo (4,5-F) Quinoxaline
DNA: Deoxyribonucleic Acid
EPIC: The European Prospective Investigation into Cancer and Nutrition
ETBF: Enterotoxigenic Bacteroides Fragilis
FadA: Fusobacterium Adhesin A
FMO3: Flavin Monooxygenase 3
Gal-GalNAc: D-Galactose-Β (1–3)-N-Acetyl-D-Galactosamine
HCA: Heterocyclic Amines
IARC: International Agency for Research on Cancer
IFN-γ: Interferon Gamma
IL-1β: Interleukin 1 Beta
IL-6: Interleukin 6
IL-8: Interleukin 8
IL 17: Interleukin-17
KRAS: Kirsten Rat Sarcoma Virus
M3R: Muscarinic Receptor
MAPK: Mitogen-Activated Protein Kinase
MIR21: MicroRNA21
MUC2: Mucin 2
MUC5AC: Mucin 5AC
NF-κB: Nuclear Factor Kappa
NLPP3: NOD-Like Receptor Family Pyrin Domain Containing 3
NOC: N-Nitroso Compounds
PAH: Polycyclic Aromatic Hydrocarbons
PI3K: Phosphoinositide 3-Kinases
PGE2: Prostaglandin E2
PKS: Polyketide Synthase
ROS: Reactive Oxygen Species
RNS: Reactive Nitrogen Species
TH17: T Helper 17 Cells
TMAO: Trimethylamine-N-oxide
TGF-β: Transforming Growth Factor Beta
TLR2: Toll-Like Receptor 2
TNF-α: Tumor Necrosis Factorα
TP53: Tumor Suppressor Gene
